# 

**Published:** 1894-12

**Authors:** 


### CONTENTS.

| | PAGE |
|---|---|
| **Comparative Pathology.** By A. J. Harrison, M.B. Lond. ... ... | 273 |
| **Cases of Senile Nerve Degeneration Resembling General Paralysis.** By S. W. MacIlwaine, M.R.C.S. Eng., L.R.C.P. Lond. ... ... | 294 |
| **Progress of the Medical Sciences:** | |
| **Medicine.** By R. Shingleton Smith, M.D. ... ... ... | 297 |
| **Surgery.** By James Swain, M.D. | 302 |
| **Progress of the Medical Sciences:** | |
| **Laryngology.** By Barclay J. Baron, M.B. ... ... ... ... | 306 |
| **Reviews of Books** ... ... ... | 309 |
| **Notes on Preparations for the Sick** | 326 |
| **Meetings of Societies** ... ... ... | 329 |
| **The Library of the Bristol Medico-Chirurgical Society** ... ... | 330 |
| **Scraps** ... ... ... ... ... | 335 |

